# ‘Am I ever going to get back to being how I was before?’: the experience of emergency laparotomy for older people living with frailty

**DOI:** 10.1186/s12877-025-06701-2

**Published:** 2025-12-30

**Authors:** Angeline Price, L Pearce, J.A Smith, P Martin, L Tomkow, J Griffiths

**Affiliations:** 1 Advanced Practitioner, Department of Ageing and Complex Medicine, Salford Care Organisation, Salford, UK; 2 Department of Nursing, Midwifery and Social Work, University of Manchester, Manchester, UK; 3 Department of General Surgery, Salford Care Organisation, Salford, UK; 4 Department of Psychological Science, Birkbeck, University of London, London, UK; 5 Department of Applied Health Research, University College London, London, UK; 6 Division of Population Health, Health Services Research & Primary Care, University of Manchester, Manchester, UK; 7 Department of Nursing, Midwifery and Social Work, University of Manchester, Manchester, UK; 8 Department of Ageing and Complex Medicine, Salford Royal Hospital, Stott Lane, Salford, M6 8HD UK

**Keywords:** Frailty, Emergency surgery, Laparotomy, Lived experience, Qualitative

## Abstract

**Introduction:**

Older people living with frailty are at high risk of adverse clinical outcomes following emergency laparotomy, including functional deterioration, hospital readmission, and death. Despite this, there is a paucity of literature exploring patient experience in this group, and little is known about what factors influence recovery. As a result, there is limited information to guide the development of robust post-operative care pathways that support optimal recovery and improve the overall experience.

**Methods:**

Twenty older people, aged ≥ 65 years, with a Clinical Frailty Scale score of ≥ 4 and who had undergone emergency laparotomy were recruited from eight hospital sites over an eight-month period. Semi-structured interviews were undertaken approximately one month after surgery to explore the peri-operative and early recovery experience. Data were analysed using reflexive thematic analysis.

**Results:**

Participants described their experience of undergoing emergency laparotomy over five temporal themes, starting at the experience around the time of surgery, followed by the early recovery period and ending with reflections of the overall experience: *feeling out of control in the acute phase*,* memory and understanding of the surgery*,* physical and psychological implications*,* transitional care needs*,* reflecting on recovery*.

**Conclusion:**

Undergoing emergency laparotomy appears to be a significant and potentially life-changing event for older people living with frailty, but one that they expressed gratitude to have experienced to remain alive. Our findings highlight the challenges encountered by this group across the perioperative and early recovery period, indicating that adaptations to service delivery may improve this experience and facilitate recovery.

**Supplementary Information:**

The online version contains supplementary material available at 10.1186/s12877-025-06701-2.

## Background

Around 30,000 people undergo emergency laparotomy each year in the United Kingdom (UK), with over half of these aged 65 years or above [1, 2]. Over the past decade, targeted national improvement initiatives, including the National Emergency Laparotomy Audit (NELA), have aimed to standardise practice and improve care for this group of patients [1, 3, 4]. Since the publication of the first annual report, NELA has demonstrated a steady reduction in overall 30-day mortality from 11.7% in 2015 to 8.7% in 2021 [5, 6]. Although the trend towards reduced mortality is most evident in patients aged 65 years or older, outcomes following emergency laparotomy remain significantly worse for older people in comparison to younger age groups [5].

The association between frailty and adverse clinical outcomes following emergency laparotomy is well established. Frailty is a multidimensional clinical syndrome characterised by age-associated decline in physical and cognitive health, and exists on a continuum ranging from robust health to severe vulnerability [7, 8]. The Clinical Frailty Scale (CFS) is a widely recognised frailty identification tool, comprised of a 9-point numerical system which is used to assess an individual’s level of frailty based on physical functioning, comorbidities, and cognitive health [8]. A score of 1 represents the most robust individuals, through to 9, which represents those who are terminally ill and expected to live less than six months. CFS 4 represents a critical transition point, corresponding with the presence of very mild frailty.

Among older adults, those living with frailty are at greater risk of postoperative complications, prolonged hospital admission, and increased care requirements upon discharge [5, 9–13]. In the 2021 NELA report, 30-day mortality in those living with frailty was 18.6%, more than double the national average [5]. Long-term clinical outcomes have been less extensively studied [14], but evidence suggests that frailty is associated with increased functional dependence and death up to 12 months following surgery [15–19]. Hospital readmission rates in those living with frailty are also higher, with fewer days spent alive and at home than experienced by non-frail patients [15, 17, 20].

Recent guidance has aimed to address disparities in emergency laparotomy care to improve patient outcomes [4, 21, 22]. However, this focuses predominantly on pre- and peri-operative elements of care, with fewer recommendations to support post-operative care delivery. A recognised limitation is the paucity of available evidence in this area, and the needs of older people living with frailty have been identified as a particular challenge [22]. Despite the growing body of quantitative literature, only a handful of qualitative studies have explored patient experience of emergency laparotomy [23–27]. Yet this approach offers potential to provide insight into how services might be delivered to best meet the needs and expectations of this patient group [28, 29].

Current available evidence highlights decision-making, provision of information, and longer-term follow-up needs as priority areas for improvement [23–25]. However, no study to date has focused specifically on the experience of older people living with frailty, despite this group being amongst the most vulnerable. It remains unclear if the experiences of older people living with frailty may differ to younger, more robust patients, or if there are specific challenges faced by this group.

Improved understanding of the recovery process from the patient’s perspective is imperative to developing perioperative care pathways that promote optimal recovery in a way that is meaningful to patients. By understanding more about postoperative care needs it may be possible to design interventions to reduce the risk of adverse outcomes for older people living with frailty [30]. This study aimed to explore the experience of emergency laparotomy for older people living with frailty, identifying any patient-reported factors that influence recovery.

## Methods

This qualitative study was undertaken as part of a broader mixed-methods PhD study, exploring the impact of emergency laparotomy for older people living with frailty. The study design was informed by Patient and Public Involvement and Engagement (PPIE) with funding from the National Institute for Health and Care Research (NIHR) Research Design Service Northwest. The doctoral student, AP, is a nurse clinical academic who has undertaken formal training in conducting research interviews and qualitative analysis. Throughout this study, AP worked in a non-clinical role and had no pre-established relationship with any potential participant. To maintain reflexivity throughout the process, AP dictated field notes to capture thoughts, emotions, and initial perceptions arising from the interviews. These were discussed with the supervisory team and revisited during data analysis, enabling AP to critique her positionality as a nurse-researcher and any assumptions that could influence the interpretation of participant accounts. Member reflection and crystallisation in the context of the overall PhD study were prioritised over individual member-checking in this study [31]. Work is underway to develop plain English summaries of the research with PPIE input. The study findings are presented here following the Consolidated criteria for Reporting Qualitative Health Research (COREQ) checklist [32] (Additional file 1).

### Design and participants

We used a semi-structured individual interview design to talk to older people living with frailty about their experience of undergoing emergency laparotomy, exploring the peri-operative and early (within 30 days) recovery phase. We aimed to develop a broad overview of a range of experiences, seeking both experiential and perspectival diversity within the data [33, 34]. For this reason, all patients who had undergone emergency laparotomy as outlined by the NELA criteria [35], were eligible for inclusion if they were aged 65 years or above and had a documented pre-operative Clinical Frailty Scale (CFS) score of 4 or more, consistent with at least a very mild degree of frailty [8]. We anticipated a sample size of 20–30 participants, based on a review of similar studies [23–25] and current literature relevant to the conceptualisation of data ‘saturation’ in reflexive thematic analysis [34, 36].The concept of ‘information power’ was used to evaluate the adequacy of the sample size throughout the data collection period, based on the aims of the study, depth of interview dialogue, and the planned analysis [36].

To increase the likelihood of diverse representation within the sample, in terms of age, frailty status, and indication for surgery, Participant Identifying Centre (PIC) sites from across the UK were invited to contribute to study recruitment. There is national variation in emergency laparotomy care delivery systems and processes; hence, the use of PIC sites enabled us to capture the experience from participants across a range of these. Thirteen PIC sites were registered, including a mix of district general hospitals and tertiary surgical centres across both inner-city and semi-rural locations in England and Scotland. A local collaborator was identified at each PIC site; all were clinicians delivering routine post-operative care to older people following emergency laparotomy.

At the host site, potential participants were identified either by the clinical team or by the primary researcher (AP) through screening of clinical notes. Those meeting the study eligibility criteria were approached by AP before discharge from the hospital. AP introduced herself and her research interests in the perioperative care of older people. If interested in receiving further information, potential participants received a patient information sheet (PIS) and a brief overview of the study. Those who remained interested provided consent to be contacted nearer to the potential interview date. At the Participant Identifying Centre (PIC) sites, local clinical collaborators identified potential participants, provided the PIS, and obtained consent for AP to contact them via telephone to discuss participation in the study. To increase accessibility to the study, all potential participants were offered the option to nominate a proxy who could facilitate the logistics of the interview and be present to provide practical support.

### Data collection

Semi-structured interviews were undertaken by AP. Participants were offered the option of either a telephone or a video interview. Those recruited from the host site were also offered a face-to-face interview. The use of telephone interviews has become more widely utilised over recent years, and there is growing evidence to suggest this method can yield data equivalent to that of a face-to-face interview if handled effectively [37, 38]. It was highlighted by the PPI group that some participants may have difficulty managing telephone interviews due to hearing loss or logistical problems. The option to have a relative or carer present to support the participant’s involvement was recommended and adopted. The initial version of the interview guide was developed by the research team (AP, JG) based on an extensive literature search and clinical experience (Additional file 2). The semi-structured nature of the interview allowed a reflexive approach to questioning, enabling important personal insights into each participant’s experience to be captured [33, 34]. Interviews were audio-recorded and transcribed verbatim, with a unique participant identifier code used for audio recording storage. To avoid overburdening participants, a decision was made not to return individual transcripts for comment or correction.

### Data analysis

The analysis was undertaken following the steps of reflexive thematic analysis [33]. This approach to thematic analysis offers flexibility in the application of underpinning theoretical assumptions. We took an inductive, critical realist approach to the analysis, identifying semantic meaning within the data [39]. This approach enabled us to interpret each participant’s account as a real and concrete experience to them, whilst remaining aware of the broader societal and cultural context that might influence this [40]. The analysis was undertaken by AP, with supervisory support from JG. It was an iterative process, beginning with familiarisation with the data through multiple readings and noting of ideas alongside review of the audio recordings. This included reviewing the transcripts for accuracy and noting any inflections or pauses in speech. Preliminary coding identified excerpts that were deemed relevant to the research question. Preliminary codes were subsequently refined by combining duplicate or closely similar codes. Code names were reviewed to ensure they accurately reflected the content of the included extracts. Extracts that did not align with the code name were recoded appropriately. Codes were then grouped into potential themes to reflect patterns across the dataset [40]. The themes were reviewed, then all transcripts were re-read to identify any previously overlooked data relevant to the themes. An example of the coding tree for the theme *‘physical and psychological implications’* is shown in Additional file 3. NVivo v12 software was used to manage data analysis [41].

## Results

Participants were ultimately recruited from eight hospital sites in England between 8th November 2022 and 15th August 2023. Five sites, including two in Scotland, were unsuccessful in recruiting any participants. Reasons included lower-than-expected numbers of older people living with frailty undergoing emergency laparotomy during the recruitment period and a lack of time and resources to comprehensively screen for potential participants. Following initial contact, four potential participants declined to take part due to ill health or burden from other clinical appointments, and three could not be contacted to arrange an interview. One potential participant living with dementia was unable to take part due to a lack of recollection of the experience at the time the interview was due.

Twenty interviews were conducted in total, in keeping with our anticipated sample size. Interviews were undertaken predominantly via telephone due to geographical constraints (telephone *n* = 15, face-to-face *n* = 5). Those undertaken face-to-face were arranged at a place and time convenient to the participant. One participant opted for their spouse to be present during the interview; the remainder were interviewed alone. The option of video calling was declined by all. Written consent was obtained before face-to-face interviews, and telephone consent was obtained prior to telephone interviews. The mean interview duration was 29 min. Participant characteristics are shown in Table 1. The mean time elapsed between the date of surgery and the interview was 43 days. One participant remained a hospital inpatient at the time of the interview; the remainder had returned home.

**Table 1:** Participants characteristics

Participant	Recruitment site	Age	Pre-operative CFS score	Operation	Time since op	Interview duration
1	Site A	68	5	Small bowel obstruction- adhesiolysis	32 days	41 min
2	Site A	78	7	Small bowel obstruction- adhesiolysis	62 days	22 min
3	Site B	74	5	Small bowel obstruction – laparoscopic adhesiolysis	22 days	18 min
4	Site C	80	5	Open subtotal colectomy/ileostomy for ischemic bowel	25 days	21 min
5	Site B	66	5	Open subtotal colectomy/ileostomy for colonic abscess	63 days	26 min
6	Site A	73	6	Small bowel obstruction- adhesiolysis	37 days	38 min
7	Site D	68	5	Open appendectomy	23 days	36 min
8	Site E	77	5	Hartmann’s, peritoneal toileting and rectal washout	96 days	18 min
9	Site F	76	5	Hartmann’s (volvulus)	35 days	24 min
10	Site D	73	6	Laparoscopic defunctioning loop sigmoid Colostomy/Repair of small paraumbilical hernia	29 days	24 min
11	Site B	88	4	Laparoscopic Hartmann’s (volvulus)	40 days	44 min
12	Site B	92	4	Repair of duodenal perforation	43 days	17 min
13	Site D	85	7	Laparoscopic adhesiolysis/reduction of small bowel.	61 days	9 min
14	Site G	68	5	Open transverse colostomy for large bowel obstruction secondary to diverticular disease	56 days	30 min
15	Site A	89	5	Large bowel obstruction – excision of mass, subsequently re-opened to re-suture	49 days	31 min
16	Site C	89	6	Gastropexy, division of adhesions, parastomal hernia repair	29 days	44 min
17	Site A	76	4	Small bowel obstruction- adhesiolysis following an elective low anterior resection	43 days	53 min
18	Site H	78	4	Untwist of jejunal volvulus plus washout	27 days	26 min
19	Site F	68	4	Small bowel obstruction- adhesiolysis	37 days	41 min
20	Site D	89	5	Small bowel obstruction- adhesiolysis	44 days	23 min

Participants discussed their experience following emergency laparotomy. Their experience is presented here across five temporal themes starting with the experience around the time of surgery, followed by the early recovery period and ending with participants’ reflections of the overall experience: *feeling out of control in the acute phase*,* memory and understanding of the surgery*,* physical and psychological implications*,* transitional care needs*,* reflecting on recovery.*

### Feeling out of control in the acute phase

Most participants described a rapid deterioration in health leading up to the surgery. The acuity of their illness and severity of symptoms invoked a negative emotional response, including shock, fear, and vulnerability.

*Erm*,* it could*,* erm… I can’t remember properly*,* but I know it was*,* er*,* it was very negative at the time*,* and a bit scary (Participant 19).*

Participants described feeling overwhelmed by the urgency of the situation, with little time to process what was happening to them. This was exacerbated by the presence of severe symptoms, including pain and vomiting. As a result, several participants described feeling unable to engage with information receiving and decision-making, and many relied on clinicians and family members to navigate the pre-operative period. Some participants described feeling completely disengaged from the situation:

*I was numb. But all I wanted was this pain to go away… (P14).*

A common perception amongst participants was that there had been no choice other than to undergo surgery, with the options perceived as being either a possibility of survival or certain death. As a result, participants had accepted the risks involved. They described taking a fatalist approach towards the outcome and adopted a sense of resignation towards their situation.

*‘Well it is a decision of two*,* innit? So you can take it and die or not take it and die*,* so…’ (Participant 8).*

In contrast, other participants described a prolonged period of symptoms, overall deterioration, and recurrent hospital admissions in the weeks to months preceding the emergency laparotomy. One participant explained how she had repeatedly sought medical input:

*Well do they think I’m pulling their legs*,* am I telling lies? … the last time I was absolutely at my wits end*,* and exhausted (Participant 16).*

For this group, the possibility of a definitive treatment was described as a blessing in disguise and a chance to regain control of their health and well-being.

*‘this was the only option that… that I could see as a way of regaining something like my former life’ (Participant 11).*

### Memory and Understanding of the surgery

Participants frequently had difficulty recollecting the details surrounding their surgery, with some unable to remember much of the perioperative period, or even their entire hospital admission.

*Well I didn’t know much about it really*,* I went in and didn’t know what was wrong*,* and I can’t remember a thing about it (Participant 4).*

Despite these gaps in memory, all participants recognised the seriousness of the circumstances they had faced, and acknowledged how close to death they had been. Several were able to describe in simple terms the reason for needing an emergency laparotomy, including a *‘squidgy’* and *‘sticky’* bowel, but others remained unsure of the exact nature of the surgery.

*Yes*,* it’s*,* er*,* it has a name*,* er*,* what did they call it*,* er*,* oh crikey*,* something I’ve never heard of before. Hold on*,* er*,* it’s an abdominal operation*,* er*,* a lapa-something or other (Participant 3).*

Notably, not all participants viewed this lack of recollection negatively, with several explicitly stating they preferred not to remember the distressing aspects of the experience:

*‘my brain if you like*,* was blanking out some of the horrible bits’ (Participant 6).*

Following the surgery, experiences of information provision varied. Participants described differences in the information they received and in the quantity and depth of information they had wanted. Some felt well-informed, describing how staff had dedicated time to answer their questions. Conversely, many participants felt that their information needs had been either overlooked or that they would have preferred to receive information in simpler terms.

In particular, discharge advice was often perceived as insufficient. Participants described feeling unsure of what information would be useful and didn’t know when or what questions to ask. Lack of detail around recovery and what to expect left them feeling unsure of how they would manage once they returned home. One participant felt it would be helpful to have a *‘list of things to look out for’ (Participant 1).*

### Physical and psychological implications

Participants described physical, psychological, and social effects following the surgery, which persisted further than hospital discharge. Participants wished they had been better counselled about the potential physical and psychological consequences of the surgery. They felt it would have been helpful to be provided with specific details on what symptoms or changes to be aware of.

Significant alterations to bowel habits were reported by almost all participants. Diarrhoea and urge incontinence were particularly problematic in the early recovery period, and diarrhoea often continued once home, hindering recovery efforts. Many wished that they had been informed more explicitly that they could experience changes to their bowel habits and provided with information on how to manage their bowels more effectively at home.

*Diarrhoea seems to be my life now*,* erm*,* even*,* I take*,* I take so much bowel meds*,* just to get going*,* and then it*,* of course it goes the other way*,* and I can’t*,* I literally can’t win with my bowels (Participant 1).*

Nutrition was a concern for many, with reduced appetite and weight loss common both before and after surgery. In the hospital, lack of appetite and poor-quality food were highlighted as barriers to eating well. Once home, several participants struggled to increase their dietary intake and described forcing themselves to eat. Finding a balance with foods that did not cause unwanted bowel symptoms was challenging and led to some frustration amongst participants and their relatives, who were keen to encourage them to eat. In contrast, several participants who had lost weight as a result of troublesome symptoms before the surgery were pleased that they were now able to eat and enjoy food without dietary restrictions.

Physical weakness was another common issue. Almost all reported some difficulties in getting up and moving following surgery. Whilst some regained their usual level of mobility whilst in hospital, several participants remained reliant on walking aids or other equipment, with some also limited by attachments including vac dressing machines and urinary catheters, once home.

*I had to walk about and that (with the physiotherapist)*,* that was*,* that was a real struggle mind… I lost all my muscles and it was difficult*,* but I had to do it*,* you know what I mean (P5).*

Several participants also discussed the emotional impact of surgery, which appeared to be linked to physical problems they were experiencing. Many reported low mood but had sense that their psychological health needs were overlooked by staff:

*I used to burst into tears*,* and the nurses would say*,* “Oh what’s the matter*,* what’s the matter? You shouldn’t be crying*,* you’re doing really well” …. Well*,* it doesn’t matter how well you are doing. you do get your down days (P19).*

Others described feeling *‘moody and quick tempered’* as a result of being unable to undertake their usual daily activities. Some viewed themselves as ‘*disabled’* and felt they were *‘rotting away’*, and wondered if they would ever return to their previous selves:

*I had some days where I felt really down and I thought*,* you know*,* ‘Am I ever going to get back to being how I was before’ But they didn’t last long (Participant 6).*

Low mood also affected concentration and motivation for several participants, but emotional support from family and friends was described as invaluable in motivating participants to persevere in their recovery.

### Transitional care needs

Physical weakness remained challenging after discharge. Participants described being *‘slowed down’* and *‘drained of energy’*, sometimes needing to spend significant amounts of time in bed during the day as a result.

*You don’t understand the tiredness*,* doesn’t matter what people say to you in the hospital*,* you do not understand the tiredness when you get home (Participant 1).*

This left many participants unable to carry out their usual day-to-day activities, including showering and dressing, but also prevented them from taking part in some social activities that they enjoyed.

*I’m dying to be out… There’s things going on downstairs [sheltered accommodation complex] that I can’t go to… I like my bingo (Participant 4).*

All participants described a reliance on family and neighbours for both practical and emotional support. Whilst in most cases family members were needed to help with tasks such as housework and shopping, several were also relied upon to support participants with washing, dressing and meal preparation. In some cases, relatives had also learned how to provide stoma care. Having family close by was deemed imperative, and participants felt they would have struggled significantly without this type of informal support. Many reflected on how they would have coped had they lived alone.

Although participants generally felt well supported by informal caregivers, some worried about the physical and emotional strain this placed on their loved ones, describing themselves as a burden and feeling guilty that relatives carried such responsibility for their care.

*Oh good God*,* yeah*,* he’s (husband) gone downhill*,* like*,* he had to go to the doctors and they put him on antidepressants… and everything like*,* and it’s a strain on him I’ve got to admit (Participant 5).*

Participants with a stoma or ongoing wound care needs received input from stoma and/or community nurses and were highly appreciative of this contact. Some participants were followed up at home by rehabilitation or therapy teams, and one participant also received input from a colorectal nurse team. These participants greatly valued being able to get in touch with someone promptly with any issues or for advice. Those who had been provided with telephone numbers of whom to contact with any problems found this reassuring, and felt it had been beneficial in reducing their anxiety around returning home.

*It makes you feel that somebody’s*,* er*,* in*,* you know*,* interested in how you’re progressing*,* er*,* which is good*,* yeah… I like the idea of speaking to somebody… I used to see in the ward (Participant 17).*

In contrast, other participants received no further input following discharge, even in some cases where this had been anticipated. Whilst some felt satisfied that no further input was required, others described feeling unsupported and *‘abandoned’*, leaving them uncertain of how to progress their recovery independently. Many were unclear on what follow-up to expect and reported difficulties in accessing advice from the General Practitioner.

### Reflecting on the experience

Despite the often-arduous surgical recovery, all participants felt grateful for having survived the operation and making progress in their recovery. Many described feeling lucky to be in their current situation, given the significance of what they had been through. Despite their circumstances, participants also displayed resilience towards their ongoing recovery, often describing themselves as determined and strong. Some used humour when reflecting on changes to their body image, such as scarring or stoma formation, whilst others regarded these changes as unnatural but accepted them as necessary to remain alive. Pre-existing health problems were also a prominent concern for many, with the emergency laparotomy viewed as one of many problems to contend with and not always their primary issue. Some displayed pragmatism towards dealing with their accumulating health issues.

Participants accepted that the recovery process would take some time and planned to approach this step by step. There was a strong sense of stoicism, with participants adapting to their new circumstances, accepting the challenges they faced, and recognising that they were still early in their recovery journey. Maintaining a positive attitude towards recovery was deemed important to participants. The main goal for most was to regain some semblance of *‘normality’* of how they had been prior to the surgery. For some, this was to be symptom-free and able to eat well. Others wanted to be able to get out in the garden, undertake daily household chores, and resume their social interactions.

All participants were satisfied with the outcome of the surgery, deeming it successful and the right course of action. None displayed regret for the decision they or others had made, although one participant reflected on the overall impact of the surgery.

*I didn’t personally think it would change my life*,* but it has. In a big way (Participant 14).*

For those who had been troubled by symptoms and deteriorating for a while leading up to surgery, the operation was viewed as *‘a life saver’*.

*Deep down I know it’s the best thing I’ve had done (Participant 9).*

## Discussion

In this study, older people living with frailty described their experience of undergoing emergency laparotomy, spanning the perioperative period and early recovery phase. These accounts provide important and novel insight into the perspectives of this specific group and the challenges they face. Recognising the distinct needs of older people living with frailty, as compared to younger, more robust groups, is crucial for driving meaningful improvement after this type of surgery. Furthermore, this work contributes to the existing literature on frailty and emergency surgery, which has been largely informed by quantitative research. By highlighting patient perspectives, it offers complementary insight that may help to inform more patient-centred approaches to care. The findings are particularly relevant given the considerable risk of adverse outcomes among older people living with frailty undergoing emergency laparotomy.

Undergoing emergency laparotomy appears to be a significant and life-altering event for older people living with frailty, associated with a range of unanticipated physical and psychological consequences. Although participants generally viewed the surgical outcome positively, the perioperative period was uniformly challenging. Reported difficulties included altered bowel functioning, nutrition, and psychological distress related to the hospitalisation, surgery, and recovery. These challenges were compounded by feelings of loss of control, both in accessing care and in decision-making processes, echoing findings from previous research with adults undergoing emergency laparotomy [23, 24, 26].

Our data also highlights tensions around shared decision-making. Although deemed the gold standard approach, our findings suggest that shared decision-making may be challenging or even undesirable to some older people living with frailty. Some participants were unable to recall details around decision-making, which may have offered psychological protection, whilst others expressed ambivalence towards receiving detailed information or being actively involved in the moment. This aligns with prior work describing variability in preferences around receiving information and involvement in decision-making among older people living with frailty [42, 43], suggesting that a one-size-fits-all approach to older people living with frailty may be problematic. Further research is needed to investigate how this group participates in shared decision-making across various clinical contexts and to identify any potential barriers or facilitators, especially where a non-operative approach may be an option.

Access to information in the postoperative period also emerged as a key challenge, despite many participants being unsure of the details of their surgery. Lack of information has been highlighted as a major source of anxiety for patients and families following emergency laparotomy, resulting in a lack of preparedness for onward care and recovery [23, 24, 27, 44]. For older people, especially, information sharing is integral to a safe transition between the hospital and community care [27, 45, 46]. Structured interventions such as counselling for patients and carers, along with written advice on the indication for and type of surgery, returning to everyday activities, recurrence risk, and warning signs [23–25, 27, 46], may improve preparedness and enable patients to self-manage more confidently [45].

Participants’ accounts also emphasised the importance of a holistic approach to postoperative support. Consistent with previous work exploring the experience of undergoing emergency laparotomy, our findings demonstrate a broader range of unanticipated physical and psychological consequences of surgery, which complicate recovery [23–25]. For older people living with frailty, however, these challenges may be particularly significant, as reduced physiological reserve and pre-existing vulnerability make it hard to adapt to and manage the demands of recovery. Participants in our study described pre-existing health concerns and symptoms in addition to their post-operative care needs. Routine frailty specialist input within the emergency laparotomy care pathway is currently recommended [4, 21], but it is inconsistently implemented [47]. There is a need for interventional research to explore whether this specialist input can facilitate improved patient experience and outcomes in the emergency laparotomy setting. Established postoperative care models from elective surgery, such as Enhanced Recovery After Surgery (ERAS) protocols and clinical nurse specialist teams, have proven beneficial to patient outcomes and experience [48, 49] and may provide useful frameworks.

Transitional care and access to support after discharge emerged as another important aspect of the experience. Many participants reported unmet needs after discharge, including physical, psychological, and informational support. Many were reliant on informal caregivers, consistent with findings from other surgical contexts [24, 25, 45, 50, 51]. However, in our cohort, carers were often older themselves and experienced negative consequences resulting from the need to provide this support. Routine discharge planning should consider what support networks are available for older people living with frailty in the community, with clear information on what is likely to be expected [21]. There is a role for third sector involvement in perioperative care pathways in addressing the psychosocial impact of emergency laparotomy. Older people living with frailty who live alone, or some distance from family, are a group for particular focus [24]. Lack of attention to transitional care needs risks leaving older people living with frailty feeling uncertain and susceptible to an acute deterioration that may contribute to the increased risk of hospital admission experienced by this group [9, 16].

Finally, despite these challenges, participants generally viewed the surgery as successful, were positive about their recovery, and adopted a pragmatic approach towards this. Our data aligns with the findings of a study which explored the experience of older people living with frailty following hip fracture, and suggests this group to be resilient and able to adapt to changing circumstances, whilst seeking to regain a sense of normality [50]. Nevertheless, proactive counselling and support appear to be an important aspect of enabling older people living with frailty to navigate recovery effectively. To maximise this support, postoperative care pathways must recognise and address individual recovery goals, ensuring care is tailored to what matters most to each person.

### Limitations

It is important to acknowledge the presence of survivorship bias in our cohort. To capture data relating to the experience of post-hospital recovery, it was necessary to speak to participants who remained alive a few weeks after the surgery. As such, it remains unknown how the experience of those who died in the early post-operative period or were too unwell to take part may differ from the experience of the participants in our study. To mitigate against survivorship bias, future studies might employ a longitudinal design, undertaking interviews both during the hospital admission and again further into the recovery period. Despite being unable to recruit participants from hospital sites outside England, the use of PIC sites enabled recruitment across a broad geographical range with a mix of inner-city and suburban regions. The recruitment challenges encountered in this study reflect the well-documented barriers to the inclusion of older people living with frailty in research [52, 53]. It is also important to acknowledge the challenge of engaging people with cognitive impairment in research. Although frailty and cognitive impairment often co-occur, those with significant cognitive difficulties were less likely to be able to participate in interviews, and their experiences may differ from the cohort included in this study. There may also have been older people living with frailty who were eligible to participate but were not identified at PIC sites, and it is unknown if their experience would have differed from those who were recruited. However, our cohort was representative of a range of frailty scores and ages. Whilst we aimed to recruit participants who had undergone emergency laparotomy for any indication, colorectal pathology was most prevalent and reflects national emergency laparotomy data. The findings are thus most applicable to this subgroup of patients. Although provisions were in place to recruit participants whose first language was not English, this was not achieved, and thus, the findings have limited applicability in this group. Social desirability bias is another potential limitation, especially given the clinical background of the interviewer. However, participants tended to acknowledge this positioning before moving on to give critical accounts of their experience. Finally, whilst it could be viewed as a limitation that family and carer experiences were not captured within this study, this design was intentional to ensure that the first-hand experience of older people living with frailty remained the focus. Given the tensions highlighted in our study, the experience of relatives and other significant others is an important area for future research.

## Conclusion and recommendations

Undergoing emergency laparotomy appears to be a significant and potentially life-changing event for older people living with frailty, but one that they expressed gratitude to have experienced to remain alive. Our findings highlight challenges encountered by this group across the perioperative and early recovery period, with opportunities to adapt services to improve this experience. There is a need for better access to information and counselling for both patients and carers around what to expect during the transition from hospital to community. There is also a need for better post-hospital support, including targeted advice around bowel management. Robust multi-disciplinary care pathways are a potential solution to addressing the holistic care needs of this group. Future work is also needed to explore how older people living with frailty can be supported to feel more in control during the perioperative period and decision-making.

## Supplementary Information

Additional file 1 : CORE-Q checklist, PDF.

Additional file 2: Interview schedule, word.

Additional file 3: Coding tree for theme ‘physical and psychological implications’.

## Data Availability

The datasets generated and analysed during this study are not publicly available due to the need to maintain participant privacy. Consent was not provided by participants to allow sharing of interview transcripts.

## Abbreviations

CFS: Clinical Frailty Scale
ERAS: Enhanced Recovery After Surgery
NELA: National Emergency Laparotomy Audit
PIC: Participant Identifying Centre
