# Supplementary material for: ‘Am I ever going to get back to being how I was before?’: the experience of emergency laparotomy for older people living with frailty
Source: BMC Geriatr. 2025 Dec 30;25:1073. doi: 10.1186/s12877-025-06701-2 (PMC12754852; doi:10.1186/s12877-025-06701-2)
Supplement: Supplementary file 2 — Additional file 2: Interview schedule, word. [file 12877_2025_6701_MOESM2_ESM.doc]

Interview Schedule

What are patients’ experiences in the month following emergency laparotomy, and what factors are perceived to influence recovery?

1. Can you tell me a bit about your recent hospital stay and operation?
2. How have things been since the operation?

- How are you feeling overall?

- Are you experiencing any pain or other symptoms?
- How is your walking? Are you managing to do the day-to-day activities you want to?
- Is there anything you are unable to do?
- How is your mood since the operation? How is your memory? Do you think these have changed at all?
- How are you with eating and drinking?
- Is there anything that we haven’t talked about that bothers you since the operation?

1. Is there anything you have found helpful during your recovery?

- Family?/social groups?/something else?

1. Is there anything that has made it harder for you to recover?
2. What do you think being fully recovered would look like to you? (i.e. is it the wound healing, leaving hospital, getting back to everyday activities, having no symptoms?)
3. If you were to explain to someone else who was going through a similar experience what it would be like after the operation, what would you say?
4. Do you feel the hospital team provided information that you wanted or needed?
5. Is there anything you think it is important to consider about recovering from this type of operation that I have not asked you about?
