# Supplementary material for: ‘Am I ever going to get back to being how I was before?’: the experience of emergency laparotomy for older people living with frailty
Source: BMC Geriatr. 2025 Dec 30;25:1073. doi: 10.1186/s12877-025-06701-2 (PMC12754852; doi:10.1186/s12877-025-06701-2)
Supplement: Supplementary file 3 — Additional file 3: Coding tree for theme ‘physical and psychological implications’. [file 12877_2025_6701_MOESM3_ESM.pdf]

Coding Tree: Physical and Psychological Implications

Physical and Psychological Implications

```
├── Physical
│   ├── Bowel functioning
│   │   ├── Diarrhoea
│   │   ├── Attempts to self-manage
│   │   └── Lack of preparedness/information
│   ├── Dietary intake
│   │   ├── Weight loss
│   │   ├── Appetite
│   │   └── Food choices
│   ├── Physical limitations
│   │   ├── Tiredness
│   │   ├── Mobility
│   │   └── Taking ownership of recovery needs
│   ├── Body image
│   │   └── Stoma
│   └── Impact of other health problems
└── Psychological
    ├── Impact on mood
    ├── Lack of motivation
    └── Family support – emotional
```
